# Regulatory T cells control toxicity in a humanized model of IL-2 therapy

**DOI:** 10.1038/s41467-017-01570-9

**Published:** 2017-11-24

**Authors:** Yan Li, Helene Strick-Marchand, Ai Ing Lim, Jiazi Ren, Guillemette Masse-Ranson, Dan Li, Gregory Jouvion, Lars Rogge, Sophie Lucas, Bin Li, James P. Di Santo

**Affiliations:** 1 Institut Pasteur, Innate Immunity Unit, Immunology Department, 75724 Paris, France; 2 Inserm U1223, 75724 Paris, France; 3 Key Laboratory of Molecular Virology and Immunology, CAS Center for Excellence in Molecular Cell Science, Unit of Molecular Immunology, Institut Pasteur of Shanghai, Shanghai Institutes for Biological Sciences, Chinese Academy of Sciences, 200025 Shanghai, China; 4 Shanghai Institute of Immunology, Shanghai JiaoTong University School of Medicine, 200025 Shanghai, China; 5 Institut Pasteur, Human Histopathology and Animal Models Unit, 75724 Paris, France; 6 Institut Pasteur, Immunoregulation Unit, Immunology Department, 75724 Paris, France; 7 de Duve Institute, Université Catholique de Louvain, and WELBIO, B1200 Brussels, Belgium

## Abstract

While patient selection and clinical management have reduced high-dose IL-2 (HDIL2) immunotherapy toxicities, the immune mechanisms that underlie HDIL2-induced morbidity remain unclear. Here we show that dose-dependent morbidity and mortality of IL-2 immunotherapy can be modeled in human immune system (HIS) mice. Depletion of human T cell subsets during the HDIL2 treatment reduces toxicity, pointing to the central function of T cells. Preferential expansion of effector T cells secondary to defective suppressive capacity of regulatory T (T_reg_) cells after HDIL2 therapy further underscores the importance of T_reg_ in the maintenance of immune tolerance. IL-2 toxicity is induced by selective depletion or inhibition of T_reg_ after LDIL2 therapy, and is ameliorated in HDIL2-treated HIS mice receiving the PIM-1 kinase inhibitor, Kaempferol. Modeling IL-2 pathophysiology in HIS mice offers a means to understand the functions of effector and regulatory T cells in immune-mediated toxicities associated with cancer immunotherapy.

## Abstract (web summary)

High dose IL-2 is a viable treatment option for cancer immune therapy, but the underlying mechanism for the accompanying undesirable morbidity is unclear. Here the authors show, using human immune system mouse models, that regulatory T cells and their functions on effector T cells are essential modulators of the related pathogenesis.

## Introduction

IL-2 was originally identified as T-cell growth factor primarily produced and consumed by activated T cells^1^. IL-2 influences multiple haematopoietic cells during immune responses and is a key regulator of immune homeostasis^2^. High-dose IL-2 (HDIL2) administration has been approved by the Food and Drug Administration in United States as a treatment for patients with a late stage metastatic melanoma or renal cell carcinoma for over 20 years^3,4^. Although the overall response rate in HDIL2-treated patients (about 16%) is not as high as those achieved using current immune-checkpoint therapies, such as anti-programmed cell death (PD)-1 (varying from 28 to 52%), about half of the patients responded to HDIL2 therapy have durable responses lasting for years that can be viewed as ‘cure’^5^. HDIL2 therapy is associated with severe toxic side effects that include hypotension, vascular leak syndrome (VLS), liver dysfunction, and neurological disorders^6^. Accordingly, HDIL2 treatment is limited to carefully selected patients with good cardiopulmonary functions, and is only performed in a small number of centers with experience in immunotherapy^6^. Overall HDIL2 side effects, however, correlate with treatment success since continued treatment with lower IL-2 doses, while alleviating side effects, also produced lower response rates^7^. Current clinical guidelines for HDIL2 therapy indicate that patients experiencing various toxicities should withdraw from treatment, thus depriving potentially curable patients of an effective treatment option. How HDIL2 toxicities relate to treatment efficacy is not understood, and a better understanding of this relationship could help improve HDIL2-based therapies.

Our ability to study HDIL2-mediated toxicity in the clinical setting is limited for several reasons: first, criteria for toxicity evaluation and specifics of administration practices of HDIL2 therapy vary in different centers^8^; second, ethical and safety concerns restrict measurements and treatments allowed for patients undergoing HDIL2; third, therapeutic agents used before and during the HDIL2 therapy for each patient could complicate the toxic effect of IL-2, hence making the comparison between different patients difficult^9^. As human IL-2 is active on mouse cells^10^, mouse models have been developed in order to better understand the mechanisms of IL-2-mediated toxicity, including VLS. Early studies suggested that T cells were critical cellular mediators of VLS^11^. Subsequently, studies using transfer of lymphokine activated killer cells and depletion of mouse lymphoid subsets, however, implicated NK cells^12–14^. Lung endothelial cells were shown to express a functional IL-2 receptor, suggesting their role in VLS initiation^15^. These studies suggest a complex etiology for VLS with the potential participation of both haematopoietic and non-haematopoietic cellular targets that create a toxic cytokine ‘milleu’ with elevated TNF and IFN-γ^16,17^. Still, the regulatory mechanisms that condition HDIL2 treatment efficacy and toxicity remain unclear.

Regulatory T (T_reg_) cells play a critical role in peripheral immune tolerance and condition effector T cell responses. Increased T_reg_ in patients undergoing HDIL2 therapy have been negatively associated with clinical response^18–20^. Consequently, current studies to improve efficacy of HDIL2 therapy have focused on suppressing T_reg_ functions and directing IL-2-induced expansion preferentially toward effector T cells^21,22^. Whether T_reg_ have any role in modulating HDIL2-induced toxicity is currently not known, although low-dose IL-2 (LDIL2) shows promise for treating autoimmune conditions including multiple sclerosis, systemic lupus erythematosus, and chronic graft vs. host disease (reviewed in ref. ^23^).

Humanized mice that harbor human genes, cells and/or tissues provide innovative pre-clinical models that can be used to model human diseases caused by infection, inflammation, cancer, and autoimmunity (reviewed in refs ^24,25^). Human immune system (HIS) mice can be robustly reconstituted with human haematopoietic lineages, and especially human T and B lymphocytes as well as innate sentinel dendritic cells and natural killer cells^25,26^. Human cytokines have been successfully expressed in HIS mice using hydrodynamic delivery of expression plasmids or genetic modification^27–29^. In this study, we use BALB/c*R* *ag2* ^−/−^ *Il2r* *g* ^−/−^ *S* *irpa* ^*NOD*^ (BRGS)-based HIS mice to understand immune mechanisms associated with beneficial and toxic side effects of IL-2 immunotherapy. Here we model human HDIL2 and LDIL2 therapy in HIS mice using hydrodynamic injection of human IL-2 expressing plasmids. HDIL2 in HIS mice leads to severe morbidity and high mortality, similar to that observed during HDIL2 therapy in humans. We also find that IL-2 induced toxicity is mediated through human T cells, and is associated with decreased T_reg_ homeostasis and function in HIS mice. Taken together, our results show that T_reg_, previously considered deleterious to HDIL2 immunotherapy, is implicated in IL-2 induced toxicity and can be considered as a target to optimize cytokine immunotherapy.

## Results

### A humanized mouse model for IL-2 induced toxicity

We utilized the technique of hydrodynamic injection of IL-2 encoding expression plasmids to generate graded doses of systemic human IL-2 in vivo. Unlike previous reports that studied the effects of IL-2 on murine targets^11–15^, we wished to dissect the roles for IL-2 on human haematopoietic cells in vivo. We and others have shown that human immune system (HIS mice) can be generated by engrafting human CD34^+^ haematopoietic stem cells in immunodeficient hosts^26–28,30,31^. We first studied IL-2 administration in non-reconstituted BALB/c*R* *ag2* ^−/−^ *Il2r* *g* ^−/−^ *S* *irpa* ^NOD^ (BRGS) recipients^26^. Cytokine levels in serum increased rapidly after IL-2 plasmid injection in a dose-dependent fashion but rapidly decayed within days; in mice receiving the highest plasmid dose (30 μg), sustained hIL-2 expression for at least 15 days was observed (Supplementary Fig. 1a).

**Fig. 1:**
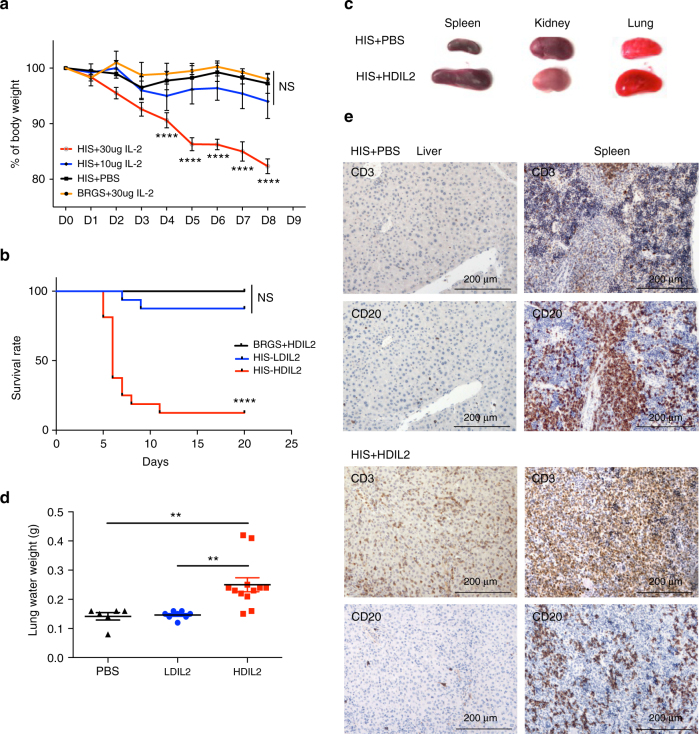
IL-2 induces dose-dependent toxicity in human immune system mice. 10–14-week-old BALB/c*R* *ag2* ^−/−^ *Il2r* *g* ^−/−^ *S* *irpa* ^NOD^ (BRGS) mice and reconstituted human immune system (HIS) mice were hydrodynamically injected with PBS only, 10 μg and 30 μg human IL-2 encoding plasmids. **a** Percentages of body weight change after hydrodynamic injection in HIS mice (*n* = 5 per group). The data are representative of two independent experiments. Comparisons are between PBS-injected HIS mice and the other groups by repeated-measures two-way ANOVA with a Sidak test. **b** Kaplan−Meier survival curves of HIS mice and BRGS mice injected with IL-2 encoding plasmids (*n* = 10 for BRGS + HDIL2 group, *n* = 16 for other groups), Log-rank test. **c** Representative images of post-mortem examination of spleen, kidney, and lung from HIS mice treated with PBS or high-dose IL-2 (HDIL2). **d** Pulmonary edema in HIS mice treated with PBS, low-dose IL-2 (LDIL2), or HDIL2 were measured by lung water weight. Lung water weight was calculated by subtracting weights of dried lungs from that of wet lungs. The data are pooled from two independent experiments, analyzed by 1-way ANOVA test with a Tukey test. **e** Representative immunohistochemistry staining of CD3 or CD20 on sections from liver and spleen of HIS mice treated with PBS or HDIL2. **P < 0.01, ****P < 0.0001; NS, not significant

We next performed hydrodynamic injection of IL-2 plasmids in reconstituted BRGS HIS mice comparing ‘high dose’ (30 μg plasmid; HDIL2) and a ‘low dose’ (10 μg plasmid; LDIL2). As expected, hIL-2 levels were increased in a dose-dependent fashion and rapidly decayed (Supplementary Fig. 1b). While LDIL2 was well tolerated, HIS mice receiving HDIL2 showed clinical signs of toxicity with loss of body weight (BW), reduced mobility, ruffled fur, and developed loose stools; HDIL2 HIS mice were killed between day 5 and day 8 when the BW loss exceeded 15% (Fig. 1a, b). By contrast, most LDIL2 HIS mice and all HDIL2 non-humanized BRGS mice had stable BW and absence of physical distress (Fig. 1a, b), indicating that human cells were necessary for the hIL-2-induced toxic effects. These data show that clinical signs of IL-2 toxicity can be observed in HIS mice in the context of sustained, elevated hIL-2 levels that mirrors the situation observed in patients receiving IL-2 immunotherapy^6,7^.

Starting at 5 days post-hIL-2 injection, HDIL2-treated HIS mice showed marked splenomegaly, nephrotoxicity, and pulmonary edema (Fig. 1c, d); these pathological changes have been previously reported in humans treated with high doses of IL-2^9^. Immunohistochemical analysis of PBS-injected HIS mice showed scant human T cells in the liver and normal lymphoid follicles in spleen with clearly defined CD20^+^ B and CD3^+^ T cell (Fig. 1e). In contrast, tissues from HDIL2-treated HIS mice showed massive expansion of T cell zones in these tissues. IL-2 is a known growth factor for T cells and we found that human IL-2 levels were strongly reduced in HIS mice compared with non-reconstituted BRGS mice (Supplementary Fig. 1b), consistent with IL-2 consumption by human immune cells.

HDIL2 toxicity in humans includes VLS manifested by pronounced tissue edema, with development of ascites, pleural effusions and pulmonary edema^16^. All HIS mice undergoing HDIL2 therapy showed signs of pulmonary edema. As an index for VLS, we quantitated lung water weight in PBS-, LDIL2-, and HDIL2-treated HIS mice (Fig. 1d); increases were observed after high-dose hIL-2 therapy. As BRGS hosts are unable to respond to IL-2, the mechanism of VLS observed following HDIL2 therapy implies a direct effect of IL-2 activated human immune cells.

Previous studies in mice investigated the nature of the target cells during IL-2 administration and suggested that both haematopoietic and non-haematopoietic (lung parenchymal cells, especially endothelial cells) might be involved^11–15^. To further explore the effects of HDIL2 in mice, we performed experiments in C57BL/6 mice, *Rag2* ^−/−^ mice (lacking B, T cells but retaining NK cells and innate lymphoid cells (ILC)), *Rag2* ^−/−^/*Il15* ^−/−^ mice (lacking B, T, and NK cells), *Rag2* ^−/−^/*Il15* ^−/−^ mice treated with anti-Thy1 antibodies (to further deplete residual NK cells and ILCs) and *Rag2* ^−/−^/*Il2rg* ^−/−^ mice (lacking B, T, NK, and ILCs) (Supplementary Fig. 1c). All of these mice express *Il2rg* on non-haematopoietic cells with the exception of *Rag2* ^−/−^/*Il2rg* ^−/−^ mice. Toxic effects were obvious in HDIL2-treated mice when NK cells and ILCs were present. However, mice lacking B, T, NK, cells and ILCs but retaining *Il2rg* expression on non-haematopoietic cells (*Rag2* ^−/−^/*Il15* ^−/−^ mice treated with anti-Thy1 antibodies) did not show toxic effects, thereby demonstrating that IL-2 acting on lung endothelial cells is not sufficient to explain IL-2 toxicity in this model.

### Systemic activation of human lymphocytes by HDIL2

**Fig. 2:**
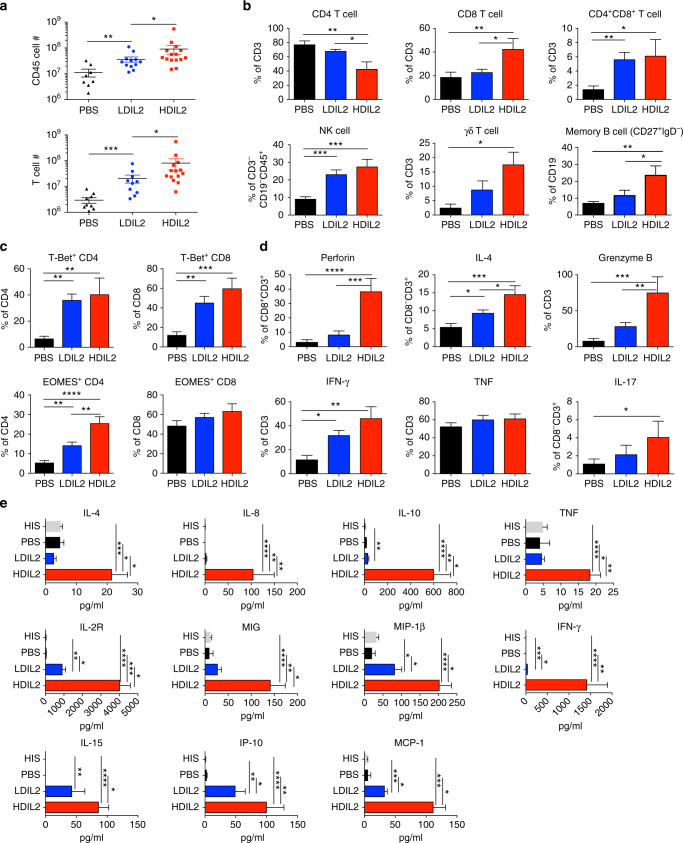
Systemic activation of human immune responses in high-dose IL-2 human immune system mice. 10−14-week-old human immune system (HIS) mice treated with PBS, low-dose IL-2 (LDIL2) or high-dose IL-2 (HDIL2) were killed at day 6 post injection. Sera were collected at day 6 post injection. **a** Absolute cell numbers ± SEM for human CD45^+^ and CD3^+^ cells in spleen of HIS mice treated with PBS, LDIL2 or HDIL2. The data are pooled from four independent experiments. **b**−**d** Percentages ± SEM of indicated human immune subsets **b**, transcriptional factor expression within T cell subsets **c**, and cytokine producing T cells **d** from spleens of HIS mice treated with PBS, LDIL2, or HDIL2. The data are pooled from two independent experiments, analyzed by 1-way ANOVA test with a Tukey test. **e** Serum cytokine levels were analyzed from non-treated HIS mice and HIS mice treated with PBS, LDIL2, or HDIL2. The data are pooled from four independent experiments, and analyzed by 1-way ANOVA test with a Tukey test. *P < 0.05, **P < 0.01, ***P < 0.001, ****P < 0.0001

Absolute numbers of human CD45^+^ cells in HIS mice increased in an IL-2 dose-dependent fashion that was largely due to an expansion of the human T cell compartment (Fig. 2a). Higher percentages of human CD4^+^CD8α^+^, CD8α^+^ T cells, TCRγδ^+^ T cells, CD56^+^ NK cells and memory B cells were observed in the spleens after HDIL2 (Fig. 2b; Supplementary Fig. 2a). IL-2 can potently expand activated and memory T cells (reviewed in ref. ^2^). We observed that a small fraction of splenic CD4^+^ and CD8^+^ T cells in HIS mice express the transcription factors T-BET and EOMES; these cells were increased in frequency in mice receiving human IL-2 (Fig. 2c). Unlike T cells from PBS-treated HIS mice, ex vivo splenic CD8^+^ T cells in HDIL2 HIS mice appeared strongly activated, expressing both perforin and granzyme B, whereas a subset of CD4^+^ T cells were either granzyme B^+^ or IL-4^+^ even in the absence of exogenous stimulation (Fig. 2d; Supplementary Fig. 2b). After stimulation, T cells from HDIL2 HIS mice produced IL-17A, and high levels of TNF and IFN-γ compared with PBS-treated HIS mice (Fig. 2d; Supplementary Fig. 2c). Moreover, both CD4^+^ and CD8^+^ T cells in HDIL2-treated HIS mice abundantly expressed HLA class II molecules and PD-1, and were largely CD45RA^–^ (Supplementary Fig. 2d). These data suggest that LDIL2 and HDIL2 therapy primarily expands pre-existing activated and memory human T cells in HIS mice.

LDIL2-activated and HDIL2-activated human haematopoietic cells appeared to be potent effector cells, as reflected by the range and amount of diverse cytokines and chemokines that were detected in the serum of HDIL2-treated animals (Fig. 2e). In contrast, PBS-treated HIS mice as well as control HIS mice receiving hydrodynamic injection of 10 μg or 30 μg ‘empty’ plasmid did not show signs of toxicity (change in BW, lung water weight) or show elevated numbers of activated T cells or cytokines (Supplementary Fig. 2e−h). A similar ‘cytokine storm’ syndrome has been reported in patients receiving high-dose IL-2 therapy^8^. Of note, many of these cytokines (including TNF, IL-4, IL-8, and MIG-1) were not induced in LDIL2 HIS mice (Fig. 2e).

### Central role of human T cells in HDIL2-induced toxicity

**Fig. 3:**
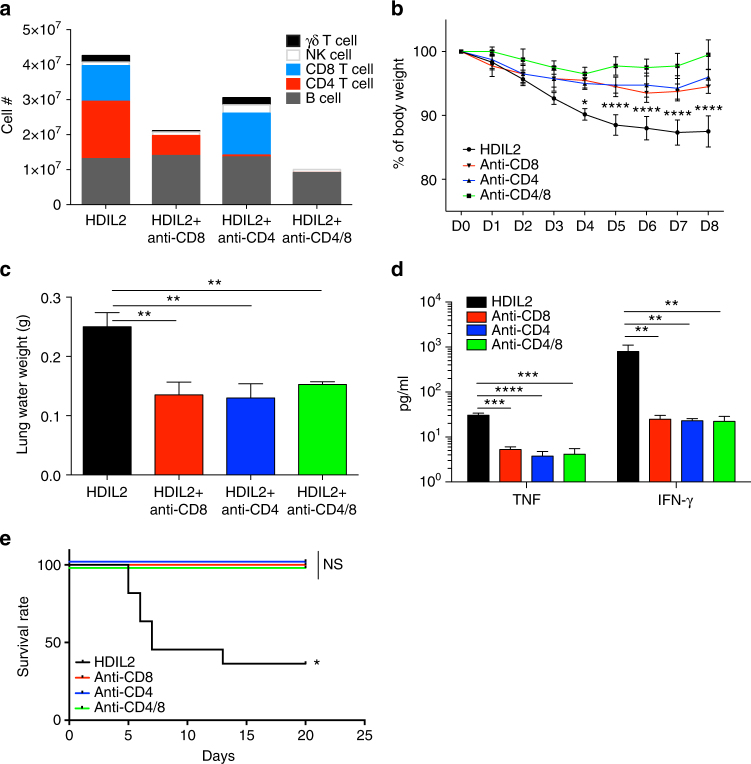
Both human CD4^+^ and CD8^+^ T cells contribute to high-dose IL-2 induced toxicity. 10−14-week-old human immune system (HIS) mice treated with PBS or high-dose IL-2 (HDIL2) (*n* = 5 per group) were killed at D8 post injection. HDIL2 HIS mice received mAbs against CD4, CD8, or CD4 and CD8 at D-1 and D3 post IL-2 treatment. The data are pooled from two independent experiments. **a** Absolute cell counts of human immune cell subsets in spleen at D8 post IL-2 treatment. **b** Body weight change after HDIL2 and mAb depletion. The data are representative of two independent experiments. Comparisons are between HDIL2 HIS mice and the other groups by repeated-measures two-way ANOVA with a Sidak test. **c** Pulmonary edema was measured by lung water weight at D8 post IL-2 treatment. The data are analyzed by 1-way ANOVA test with a Tukey test. **d** Levels of cytokine TNF and IFN-γ were analyzed from sera of indicated groups by 1-way ANOVA test with a Tukey test. **e** Kaplan−Meier survival curves of indicated group (*n* = 10 per group were analyzed by Log-rank test. *P < 0.05, **P < 0.01, ***P < 0.001, ****P < 0.0001; NS, not significant

We next treated control or HDIL2 HIS mice with mAbs that deplete CD4^+^ or CD8^+^ T cells (Supplementary Fig. 3a). The mice were observed for clinical signs, BW was measured daily and all mice were killed at day 8 post injection, when absolute cell numbers of splenic human CD4^+^CD3^+^, CD8α^+^CD3^+^, TCRγδ^+^CD3^+^ T cells, CD56^+^CD3^–^ NK cells and CD19^+^ B cells were enumerated. As expected, treatment with anti-CD4 or anti-CD8 mAbs specifically depleted the corresponding human T cell subsets (Fig. 3a). Interestingly, depletion of CD8α also led to a considerable decrease in the absolute numbers of TCRγδ T cells (Fig. 3a). The decrease of TCRγδ T cells can be explained as these cells are CD8α^+^ in HDIL2 HIS mice (Supplementary Fig. 3b). Absolute numbers of CD19^+^ B cells were not affected by T cell depletion while the numbers of CD56^+^CD3^–^ NK cells increased (Fig. 3a). Depletion of CD4^+^ T cells, CD8^+^ T cells, or both T cell subsets reduced the loss of BW observed in HIS mice receiving HDIL2 therapy (Fig. 3b). In accordance with the improved clinical picture, T cell depletion completely abolished HDIL2-induced pulmonary edema (Fig. 3c). Elevated levels of inflammatory cytokines that are associated to VLS were also lowered by depletion of CD4^+^ T cells or CD8^+^ T cells (Fig. 3d). In an independent set of experiments, we found that increased mortality associated with HDIL2 therapy was reduced in T cell-depleted HIS mice (Fig. 3e). Collectively, these results demonstrate that both CD4^+^ and CD8^+^ T cells are critical mediators of IL-2 induced toxicity.

### Reduced regulatory T cell function in HDIL2 HIS mice

**Fig. 4:**
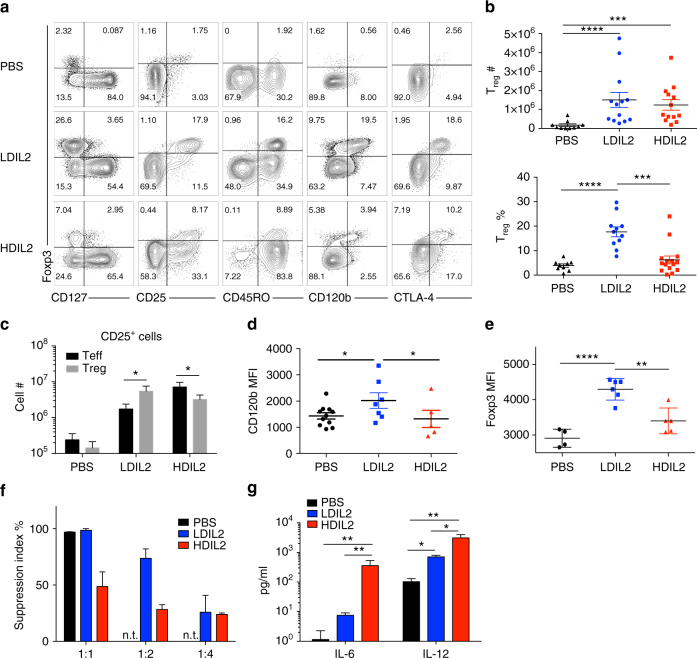
Defective regulatory T cell function in high-dose IL-2 human immune system mice. 10−14-week-old human immune system (HIS) mice were injected with PBS, low-dose IL-2 (LDIL2) or high-dose IL-2 (HDIL2). Mice were killed at D6 post injection. **a** Representative flow cytometric data showing the phenotypes of splenic Foxp3^+^ regulatory T (T_reg_) cells. The data are representative of two independent experiments. **b** Percentages and numbers of Foxp3^+^ T_reg_ cells within splenic CD45^+^CD3^+^CD4^+^ T cells. The data are pooled from four independent experiments. **c** Absolute cell counts of CD25^+^ cells within CD45^+^CD3^+^CD4^+^Foxp3^+^ T_reg_ and CD45^+^CD3^+^CD4^+^Foxp3^−^ effector T (T_eff_) cells. **d**, **e** Mean fluorescence intensity (MFI) ± SEM for CD120b and Foxp3 in CD3^+^CD4^+^CD127^−^Foxp3^+^ T_reg_ cells. The data are representative of four independent experiments. **f** CD45^+^CD3^+^CD4^+^CD25^high^CD127^−^ T_regs_ were sorted and mixed with CFSE labeled CD45^+^CD3^+^CD4^+^CD25^−^CD127^+^ T_eff_ cells at the ratio of 1:1, 1:2, and 1:4 in the presence of Dynabeads at a cell to beads ration of 1:8. The suppression index is calculated as [100 × (1−%CFSE^low^ T_eff_ cells in coculture/%CFSE^low^ T_eff_ alone)]. NT = not tested. The data are the representative of two independent experiments. **g** Levels of cytokine IL-6 and IL-12 were analyzed from sera of indicated groups. The data are pooled from four independent experiments. *P < 0.05, **P < 0.01, ***P < 0.001, ****P < 0.0001 by 1-way ANOVA test with a Tukey test

Regulatory T cells (T_reg_) are Foxp3^+^ CD4^+^ T cells that play critical roles in immune homeostasis. T_reg_ constitutively express CD25 that allows them to efficiently capture IL-2 in vivo and as such, T_reg_ numbers are correlated with IL-2 availability^32^. Previous studies have characterized T_reg_ development and function in HIS mice^33,34^. As expected, we found low percentages of Foxp3^+^ T_reg_ (3.8 ± 2.1%) in the spleens of PBS-treated HIS mice (Fig. 4a) that contrasts with higher percentages (5–9%) that comprise normal human peripheral blood^34^. After LDIL2 therapy, T_reg_ percentages were increased (17.7 ± 6.7%) consistent with a role for IL-2 in T_reg_ expansion. Surprisingly, as IL-2 was made more available in vivo (HDIL2 therapy), T_reg_ percentages were decreased (6.2 ± 6.2%). When calculating absolute numbers of T_reg_, we found no differences between LDIL2- and HDIL2-treated HIS mice, although both had more T_reg_ than PBS-treated HIS mice controls (Fig. 4b). The relative reduction in the percentage of T_reg_ in HDIL2 results from a massive increase in Foxp3^–^ cells within the CD4^+^ T cell compartment. This is also evident when examining CD25^+^ T cells in LDIL2 and HDIL2 HIS mice. T_reg_ exceed T_eff_ under LDIL2 therapy, whereas T_eff_ predominate over T_reg_ in HDIL2 HIS mice (Fig. 4c), pointing to a potential loss of T_reg_ function in this context. The difference in Treg induction between LDIL2 and HDIL2 was not due to differences in total quantity of plasmid injected (10 μg vs. 30 μg hIL-2 encoding plasmids) but rather reflects the resultant IL-2 dose (Supplementary Fig. 4).

Foxp3^+^ T_reg_ in PBS-, LDIL2-, and HDIL2-treated HIS mice had the characteristic CD127^–^CD25^high^CD45RO^+^ phenotype (Fig. 4a). The majority of T_reg_ expanded by LDIL2 expressed CTLA-4 and CD120b (Fig. 4d), indicating functional maturity^35,36^. In contrast, T_reg_ in HDIL2 HIS mice showed CD120b downregulation (Fig. 4a, d). Similarly, intensity of Foxp3 expression (MFI) on T_reg_ from HDIL2 HIS mice was decreased (Fig. 4e) suggesting compromised T_reg_ function. To verify this, we sorted T_reg_ and assessed their suppressive activity against T_eff_ cells from the same HIS mice (Fig. 4f). T_reg_ from either PBS or LDIL2 HIS mice exhibited strong suppression (Fig. 4f), while T_reg_ from HDIL2 HIS mice showed lower suppressive activity. As inflammatory cytokines, such as IL-6 and IL-12 have been reported to reduce Foxp3 expression and T_reg_ function^37,38^, we assessed levels of these cytokines in the serum of PBS or IL-2-treated HIS mice. While IL-6 and IL-12 levels were moderately increased in LDIL2 mice (6.6-fold and 6.9-fold, respectively) (Fig. 4g), levels of these inflammatory cytokines were dramatically increased in HDIL2 HIS mice (314.7-fold and 30.3-fold, respectively) and clearly above the EC50 for these cytokines (50 pg ml^−1^ and 3 ng ml^−1^, respectively) suggesting that they would have stimulatory effects on their relevant targets. In summary, HDIL2-treated HIS mice show compromised T_reg_ homeostasis and function in the context of enhanced inflammation.

### Development of VLS in LDIL2 HIS mice after T_reg_ depletion

**Fig. 5:**
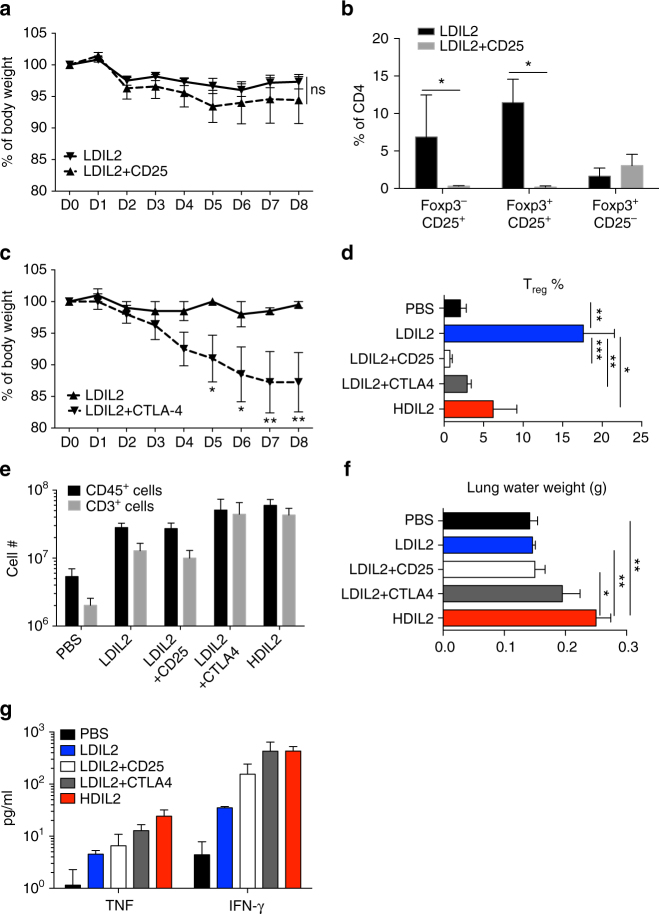
Regulatory T cell depletion in low-dose IL-2 human immune system induces toxicity. 10−14-week-old human immune system (HIS) mice treated with PBS, low-dose IL-2 (LDIL2) or LDIL2 plus anti-CD25 or anti-CTLA-4 depleting mAbs were killed at D8 post injection for analysis. Two injections of 300 μg anti-CD25 or anti-CTLA-4 depleting mAbs were given at D0 and D4. **a**, **c** Percentages of body weight change after LDIL2 treatment and mAb depletion in HIS mice were measured daily (*n* = 5 per group). The data are representative of two independent experiments. Comparison is between LDIL2 HIS mice and other groups by repeated-measures two-way ANOVA with a Sidak test. **b** Percentages of Foxp3 and CD25 expressing cells within splenic CD45^+^CD3^+^CD4^+^ T cells. **d** Percentages of Foxp3^+^ cells within splenic CD45^+^CD3^+^CD4^+^ T cells. **e** Absolute cell counts of total human CD45^+^ cells and CD3^+^ T cells in spleens of indicated groups. **f** Pulmonary edema in HIS mice were measured by lung water weight. **g** TNF and IFN-γ levels were analyzed from sera of indicated groups. The data are pooled from two independent experiments and analyzed by 1-way ANOVA test with a Tukey test. *P < 0.05, **P < 0.01, ***P < 0.001; NS, not significant

Since T_reg_ activity was compromised in HDIL2 HIS mice but intact in LDIL2 HIS mice, we next assessed a direct link between the T_reg_ immune suppression and IL-2 clinical toxicity. An anti-CD25 mAb was able to efficiently deplete T_reg_ in HIS mice (Supplementary Fig. 5a, b). We then studied the clinical effects of anti-CD25 mAb in LDIL2-treated mice. Anti-CD25 treatment was well tolerated, although slight BW loss was noted compared to control mAb injected mice (Fig. 5a), suggesting perturbed immune homeostasis.

Since considerable numbers of activated human T and NK cells express CD25^32,39^, this depletion protocol may also affect non-T_reg_ activities. Indeed, spleens of LDIL2 HIS mice after injection of CD25 mAb showed loss of both T_reg_ (Foxp3^+^CD25^+^) and activated T_eff_ (Foxp3^−^CD25^+^; Fig. 5b). We therefore targeted cytotoxic T lymphocyte-associated antigen (CTLA)-4^+^ cells (which are largely Foxp3^+^; Fig. 4a) in order to selectively deplete T_reg_ in HIS mice. Administration of anti-CTLA-4 mAb to LDIL2 HIS mice reduced circulating T_reg_ (Supplementary Fig. 5c) and provoked sustained weight loss (Fig. 5c). In contrast, the same protocol of anti-CTLA-4 mAb treatment caused neither BW drop nor lung edema in HIS mice (Supplementary Fig. 5d, e).

Both anti-CD25 and anti-CTLA-4 treated LDIL2 mice showed a reduction in the percentage of splenic Foxp3^+^ T_reg_ (Fig. 5d). When comparing the absolute numbers of human CD45^+^ cells and CD3^+^ cells in the spleen of treated mice, we found that T_reg_ depletion by anti-CTLA-4 clearly stimulated human T cell numbers in LDIL2 HIS mice (Fig. 5e) suggesting a loss of immune homeostasis secondary to T_reg_ depletion. In contrast, anti-CD25 mAb treatment did not cause changes in T cell numbers comparing to control mAb-treated LDIL2 HIS mice (Fig. 5e), possibly due to in vivo removal of CD25^+^ effector cells (such as Foxp3^–^CD25^+^ T cells, Supplementary Fig. 5b). Consistent with these hypotheses, the percentages of HLA-DR^+^ CD4 T cells are lower in LDIL2 HIS mice and anti-CD25 treated LDIL2 HIS mice than those in HDIL2 HIS mice and anti-CTLA-4 treated LDIL2 HIS mice (Supplementary Fig. 5f). Lung edema developed in anti-CTLA-4 treated LDIL2 HIS mice similar to that observed in HDIL2 HIS mice (Fig. 5f), while VLS was not observed in anti-CD25 treated LDIL2 HIS mice. Moreover, treatment with either anti-CD25 or anti-CTLA increased serum TNF and IFN-γ levels in LDIL2 HIS mice, although the latter induced much higher levels (Fig. 5g). Collectively, these results demonstrate the important role for T_reg_ function for preventing immune system activation and VLS during IL-2 immunotherapy.

### Development of VLS in LDIL2 HIS mice after T_reg_ inhibition

**Fig. 6:**
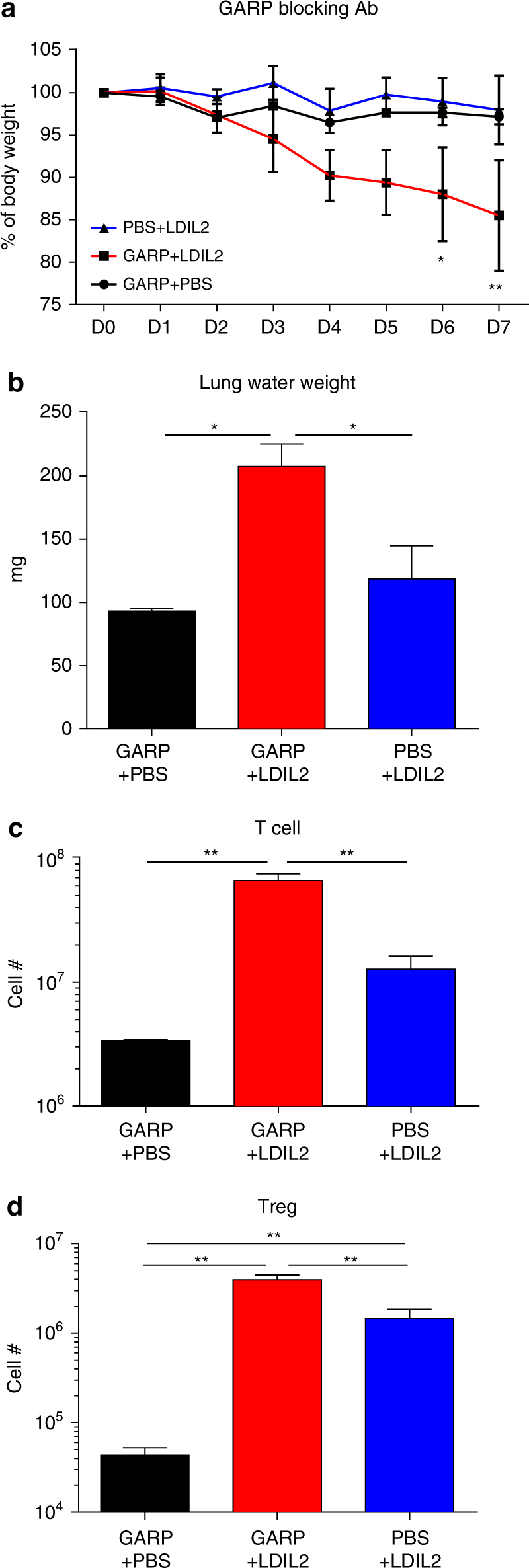
Blocking regulatory T cell function by anti-GARP mAb in low-dose IL-2 human immune system mice induces toxicity. 10−14-week-old human immune system (HIS) mice were injected i.p. with anti-GARP mAbs 100 μg daily starting from D0. At D1, 10 μg IL-2 encoding plasmids were injected hydrodynamically into low-dose IL-2 (LDIL2) treatment group. Mice were killed on D7. **a** Percentages of body weight change of indicated groups were measured daily (*n* = 5 per group). The data are representative of 2 independent experiments. Comparison is between LDIL2 treated HIS mice and other groups by repeated-measures two-way ANOVA with a Sidak test. **b** Pulmonary edema in HIS mice were measured by lung water weight. **c**, **d** Absolute cell counts ± SEM for human CD3^+^ cells and CD45^+^CD3^+^CD4^+^Foxp3^+^ cells in spleen of indicated groups. The data are representative of two independent experiments, and analyzed by 1-way ANOVA test with a Tukey test. *P < 0.05, **P < 0.01

To further investigate the role of T_reg_ in IL-2 induced toxicity, we used an anti-glycoprotein A repetitions predominant (GARP) mAb to block the suppressive function of T_reg_ without affecting the numbers of T_reg_. Membrane protein GARP is specifically expressed on T_reg_ cell surface and critical in the production of active transforming growth factor-β1 (TGF-β1), therefore serving as one therapeutic target to block T_reg_ inhibition of immune cells^40^. We tested effects of a human GARP blocking mAb (MHG-8)^40^ in LDIL2 HIS mice. While injection of GARP mAb alone or LDIL2 treatment alone in HIS mice caused neither BW drop nor lung edema (Fig. 6a, b), combining both led to severe decrease of BW and increase of lung water weight. LDIL2 HIS mice injected with GARP mAb showed loss of human T cell homeostasis compared to other groups (Fig. 6c). As GARP mAb blocks rather than depletes T_reg_, there were higher numbers of T_reg_ in this context (Fig. 6d). Hence, these data support the role for T_reg_ in controlling IL-2 induced toxicity and indicate that GARP mediated release of active TGF-β1 in T_reg_ may be involved.

### IL-2 toxicity in Kaempferol-treated HDIL2 HIS mice

**Fig. 7:**
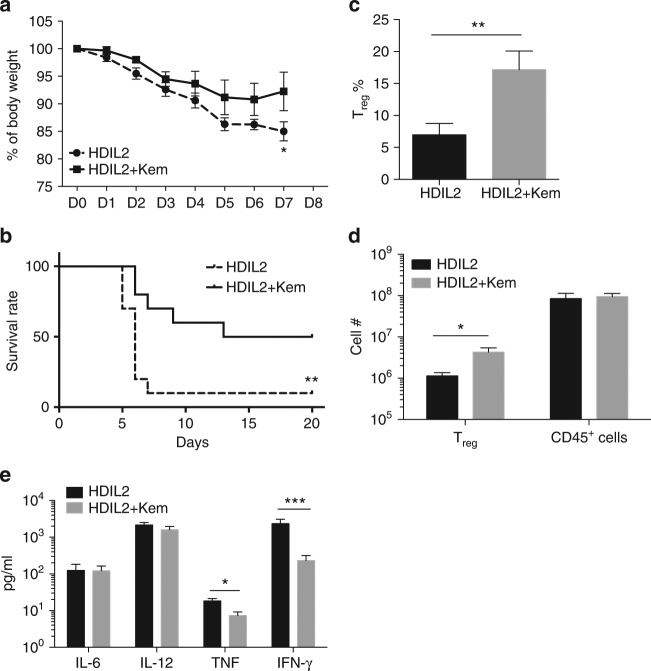
Kaempferol ameliorates high-dose IL-2 induced toxicity through regulatory T cells. High-dose IL-2 (HDIL2) human immune system (HIS) mice were analyzed at D8 after 30 μg IL-2 plasmid injection. **a** Percentages of body weight change after HDIL2 treatment with or without Kaempferol (*n* = 5 per group). The data are representative of three independent experiments. Comparison between HDIL2 HIS mice and Kaempferol-treated HDIL2 HIS mice by repeated-measures two-way ANOVA with a Sidak test. **b** Kaplan−Meier survival curves of HDIL2 HIS mice and Kaempferol-treated HDIL2 HIS mice (*n* = 12 for HDIL2 group *n* = 10 for HDIL2+ kem group) were analyzed by Log-rank test. **c** Percentages of CD25^high^CD127^−^Foxp3^+^ cells within splenic CD45^+^CD3^+^CD4^+^ T cells. **d** Absolute cell counts of human regulatory T cells and total CD45^+^ cells in spleens of indicated groups. **e** IL-6, IL-12, TNF and IFN-γ levels were analyzed from sera of HIS mice from corresponding groups. Data are pooled from three independent experiments and analyzed by Mann−Whitney test. *P < 0.05, **P < 0.01, ***P < 0.001

Activation of inflammatory pathways have been shown to lead to the post-translational modification and degradation of Foxp3 and are implicated in the loss of T_reg_-mediated suppression^37,38,41,42^. The plant flavonoid Kaempferol (kem) has been used as an anti-inflammatory agent and recently identified as a PIM-1 kinase inhibitor that can preserve T_reg_ suppressive function^43^. In vitro culture of human cord blood T_reg_ cells with kem resulted in an increase in the percentage of Foxp3^+^ CD4^+^ T cells and higher Foxp3 protein expression (Supplementary Fig. 6). Treatment of HDIL2 HIS mice with kem resulted in reduced BW loss (Fig. 7a) and improved survival (Fig. 7b). Kem treatment promoted a corresponding increase in T_reg_ frequency comparable to that observed in LDIL2 HIS mice (Fig. 7c). Absolute T_reg_ numbers were increased in kem- and HDIL2-treated HIS mice, and total CD45^+^ cell numbers were preserved (Fig. 7d). We found a decrease in TNF and IFN-γ levels in kem-treated HDIL2 HIS mice (Fig. 7e). Interestingly, IL-6 and IL-12 were not decreased by kem treatment, indicating that its effect on T_reg_ was not mediated through general inhibition of inflammation. Taken together, these data suggest that kem may prevent HDIL2-induced toxicity via restoration of T_reg_ homeostasis and function.

## Discussion

A major recent advance in the treatment of human cancer involves the therapeutic targeting of immune response ‘checkpoints’ via antibodies that interfere with cytotoxic T lymphocyte-associated antigen (CTLA)-4 and programmed cell death (PD)-1 receptors and their ligands (reviewed in ref.^44^). These two receptor systems play critical, overlapping roles in immune activation and tolerance. By blocking CTLA-4 and/or PD-1 signaling in T cells, cellular immune responses are unleashed with remarkable effects on tumor regression and long-term ‘cure’ can be achieved (reviewed in ref.^45^). Nevertheless, ‘resetting’ immune tolerance may also provoke serious side effects, and recent reports indicate that ‘checkpoint’ inhibition are often associated with immune related adverse effect, sometimes rare and lethal autoimmune phenomenon in a subset of patients^46,47^. As such, knowledge on how the human immune system maintains T cell homeostasis and tolerance becomes critical in order to optimize cellular immune responses in the absence of destructive autoimmunity.

High-dose IL-2 and ‘checkpoint’ inhibition immunotherapies share interesting properties in terms of therapeutic vs. toxic effects. Both can result in long-term clinical ‘cure’ of disease while both have dramatic treatment-dependent toxicities that arise from loss of T cell tolerance. Here we developed a HIS model that recapitulated clinical symptoms of IL-2 immunotherapy (including VLS and cytokine ‘storm’) and provides a controlled context for understanding the immune mechanisms behind therapeutic effects and toxicities. Our model identified known (IL-6, TNF) as well as potential cytokine biomarkers for VLS severity (IL-4, IL-8, and MIG-1), showing that IL-2-treated HIS mice may be useful to understand the pathophysiology of IL-2-induced toxicity, especially through targeting of human cytokines that have high species specificity^29^. It will be interesting to compare these specific biomarkers in the context of patients receiving ‘checkpoint’ inhibitors to see if they also help stratify a subset of patients with higher susceptibility to toxic effects.

IL-2 plays a critical role in the homeostasis of regulatory T (T_reg_) cells, which can potently suppress T cell responses. As such, enhancing T_reg_ homeostasis and function has long been considered as potentially deleterious in the context of anti-tumor therapies. Concerning high-dose IL-2 (HDIL2) therapy, a negative correlation between clinical outcome and percentage of T_regs_ in the blood of patients receiving treatment has been reported^18–20^. Still, these clinical studies exclude patients withdrawn from therapy due to the toxicity and focus only on the correlation between T_reg_ numbers and treatment efficacy. As a result, the relationship between toxicity, treatment efficacy and alterations in T_reg_ function has not been carefully assessed. Recent efforts to improve HDIL2 therapy include blocking IL-2 binding to T_reg_ and lung endothelial cells or use of an antibody-coupled IL-2 complexes^21,22^. These approaches will skew IL-2 triggering toward effector T cell (T_eff_) cells that should enhance therapeutic effects. Still, we show using antibody depletion that activated T cells are the major effectors of IL-2 toxicity. Therefore, achieving the proper balance between beneficial IL-2 stimulation of T_eff_ cells vs. suppressive T_reg_ conditions not only anti-tumor effects but also treatment-limiting toxicities.

IL-2 can trigger distinct classes of immune cells (T cells, B cells, myeloid cells), as well as their subsets. We found that IL-2 responses in HIS mice vary depending on IL-2 dose, suggesting threshold responses (‘all or nothing’) for certain immune cell types. For T cells, IL-2 is known to promote proliferation and activation of memory T cells^2^. We found evidence for this in HIS mice, although it is known that IL-2 can act in concert with ‘promiscuous’ TCR signals to trigger naive T cells^48^. It is possible that such effects operate during bolus infusions of IL-2 in humans and can be mimicked through hydrodynamic delivery of human IL-2 in HIS mice. It is interesting to note that patients treated with bolus IL-2 injections also showed rapid decay in vivo^23^, similar to what we observed using hydrodynamic injection.

Our results provide important new insights into the role of the cell T_eff_/T_reg_ equilibrium in IL-2-mediated toxicities. T_reg_ are preferentially expanded in low-dose IL-2 (LDIL2) HIS mice and are fully functional as shown by their ability to suppress T cell proliferation ex vivo. In contrast, T_reg_ expansion and function were compromised in HDIL2 HIS mice as a result of a pro-inflammatory environment secondary to activated Teff cells. Preliminary studies show that the HDIL2 effect was also observed in HLA-transgenic HIS mice suggesting that it was not due to aberrant T cell education. To firmly establish the central role of Treg in HDIL2-induced toxicity, we specifically depleted T_regs_ or blocked T_reg_ function in healthy LDIL2 HIS mice; this generated a toxicity similar to that observed in HDIL2 HIS mice. Moreover, we treated HDIL2 HIS mice with Chinese medicinal Kaempferol, which has recently been shown to enhance Foxp3 expression by reducing PIM-1 mediated Foxp3 phosphorylation^49^ and promote transplant tolerance by sustaining T_reg_ function^50^. While total numbers of human cells in kem-treated HDIL2 HIS mice remained elevated, levels of pro-inflammatory cytokines were substantially reduced, that may be due to expanded T_reg_ that control the cytokine ‘storm’. Still, kem may also affect T cell subsets through its suppression on DC and macrophage activation^51,52^ in addition to its role in T_reg_ modulation. Taken together, HIS mice can provide a tractable system to dissect the roles for human lymphocyte interactions during IL-2 therapy. We propose that the clinical benefit of HDIL2 (therapeutic efficacy and limited toxicity) may be determined by T_reg_ function in the context of inflammation.

## Methods

### Ethics statement

Animals were housed in isolators under specific opportunist pathogen-free/SOPF conditions with humane care and anesthesia was performed using Ketamine and Xylazine. Experiments were approved by Ethical Vigilance Committee at the Institut Pasteur (CETEA-2013-0131) and validated by the French Ministry of Education and Research (Reference #0216201).

### Generation of HIS mice

Human immune system (HIS) mice were generated in BALB/c*R* *ag2* ^−/−^ *Il2r* *g* ^−/−^ *S* *irpa* ^NOD^ (BRGS) hosts as previously described^30^. Briefly, fetal liver CD34^+^ cells were enriched (95%) using affinity columns (Miltenyi Biotec) and phenotyped for CD38 expression. Newborn (3−5 day old) mice received sub-lethal irradiation (3 Gy) by X-ray irradiator (Xstrahl Life Sciences) and were injected intrahepatically with 5 × 10^4^ CD34^+^CD38^−^ human fetal liver cells. HIS mice with less than 10% reconstitution of human CD45^+^ cells in blood at week 10 post HSC transplantation were excluded from experiments. Mice were killed by carbon dioxide asphyxiation followed by cervical dislocation at ages from 10 weeks onward.

### Flow cytometry and cell sorting

Fixable viability dye eFlour506 (ebioscience) were used to exclude dead cells. Flurochrome-conjugated antibodies from BD biosciences, Biolegend, and ebiosciences were used in dilutions according to manufactures’ instructions (Supplementary Table 1). Foxp3 staining used Foxp3/Transcription Factor Staining Buffer (ebioscience) as recommended. For intracellular cytokine detection, cells were stimulated with 50 ng ml^−1^ PMA (Sigma-Aldrich) and 1 μg ml^−1^ ionomycin (Sigma-Aldrich) for 4 h in the presence of Golgi-plug (BD biosciences) and stained using the BD cytofix/cytoperm kit (Cat ID: 554714, BD biosciences). Fortessa (BD biosciences) were used for acquisition and Flowjo (TreeStar) for analysis. Cell sorting was performed using a FACSAria II (BD biosciences). Gating strategies for flow cytometry analysis and cell sorting are detailed in Supplementary Fig. 7.

### Hydrodynamic injection

A human IL-2 cDNA clone in the vector pCMV-6-XL4 (Origene) was purified using endotoxin-free Plasmid-Maxi kit (Cat ID: 12362, Qiagen). Hydrodynamic injection was performed as described^27,28^. Briefly, 10−14-week-old HIS mice were weighed and plasmid injected i.v. (tail vein) in 1.8 ml PBS (for 20 g BW) within 7 s using 27-gauge needles.

### Quantification of lung water content during VLS

Lungs were excised, blotted to remove surface fluid, weighed in a 1.5 ml tube, dried for 72 h at 65 °C, and reweighed to determine lung water content as (wet lung weight—dry lung weight).

### Treg suppression assay

T_eff_ (CD3^+^CD4^+^CD25^−^CD127^+^) were labeled with 1 mM CFSE (Life Technologies). For suppression assay, 10^4^ T_reg_ (CD3^+^CD4^+^CD25^high^CD127^−^) were mixed with labeled T_eff_ at ratios of 1:1, 1:2, and 1:4 or T_eff_ alone. Cultures were stimulated for 72 h with anti-CD3/CD28 Dynabeads (Life Technologies) at a cell:bead ratio of 1:8. T_regs_ suppressive capacity was expressed as the relative inhibition of the percentage of CFSE^low^ cells [100 × (1−% CFSE^low^ T_eff_ cells in coculture/%CFSE^low^ T_eff_ alone)].

### Cytokine multiplex assay and ELISA

Serum was collected from 12–16-week-old HIS mice and analyzed using human cytokine magnetic 25-plex panel (Cat ID: LHC0009M, Invitrogen) on a MAGPIX machine (Luminex). Serum human IL-2 levels were measured by ELISA (MAX Deluxe kit, Cat ID: 431805, Biolegend).

### Immunohistochemistry

Tissue samples were fixed in 10% neutral buffered formalin, embedded in paraffin and 4 μm sections were cut and stained with H&E. To assess lymphocyte phenotypes, IHC analysis was performed using anti-human CD3 (clone F7.2.38) and anti-human CD20 (clone L26; both from Dako, Glostrup, Denmark) and revealed using Venatana Benchmark XT automated IHC system (Roche).

### In vivo antibody depletion assay

Anti-hCD4 (OKT4), anti-hCD8 (OKT8), anti-hCD25(7G7B6), anti-hCTLA-4 (BN13), and isotype control mIgG2a (C1.18.4) mAbs were purchased from bioXcell (UK). Anti-hGARP (MHG-8) was provided by Sophie Lucas (de Duve Institute, Belgium). Twelve-week-old HIS mice were injected with mAbs i.p. One day before and 3 days after hydrodynamic injection as indicated in Figure legends.

### Kaempferol studies

Kaempferol was kindly provided by the Shanghai R&D Center for Standardization of Traditional Chinese Medicines. HIS mice were gavaged with 2 mg Kaempferol in 100 µl propylene glycol 1 day before IL-2 hydrodynamic injection and then every 2 days afterwards. For in vitro studies, CD4^+^ T cells were sorted from cord blood and cultured in X-VIVO medium (Lonza) supplemented with 10% HI FBS, 1% GlutaMax, 1% sodium pyruvate, 1% penicillin streptomycin, 1% MEM NEAA plus 50 U ml^−1^ recombinant human IL-2 (R&D Systems) for 1-2 days. T cells were further stimulated with CD3/CD28 Dynabeads (bead: cell ratio of 1:4) in the presence of hIL-2 and/or Kaempferol before FACS analysis.

### Statistics

Statistical significance of the data were calculated using GraphPad Prism version 6 and data were depicted as mean ± s.e.m. if not stated otherwise. To compare two groups, an unpaired two-tailed Mann−Whitney *U*-test was applied. When more than two groups of samples were compared, one-way ANOVA (with Tukey’s multiple-comparison post-tests) was used. All *P*-values≤0.05 were consider significant. Estimated necessary sample sizes were biometrically determined. Batches of human CD34+ cells were randomly used for transplantation, and HIS mice were randomized into different groups. Blinding strategy was used whenever possible.

### Data availability

All the data supporting the findings of this study are available from the corresponding author upon reasonable request.

## Electronic supplementary material

Supplementary Information
