# Supplementary material for: Regulatory T cells control toxicity in a humanized model of IL-2 therapy
Source: Nat Commun. 2017 Nov 24;8:1762. doi: 10.1038/s41467-017-01570-9 (PMC5701141; doi:10.1038/s41467-017-01570-9)
Supplement: Supplementary file 1 — Supplementary Information [file 41467_2017_1570_MOESM1_ESM.pdf]

Supplementary Materials

Supplemental Figures 1-7

Supplemental Table 1

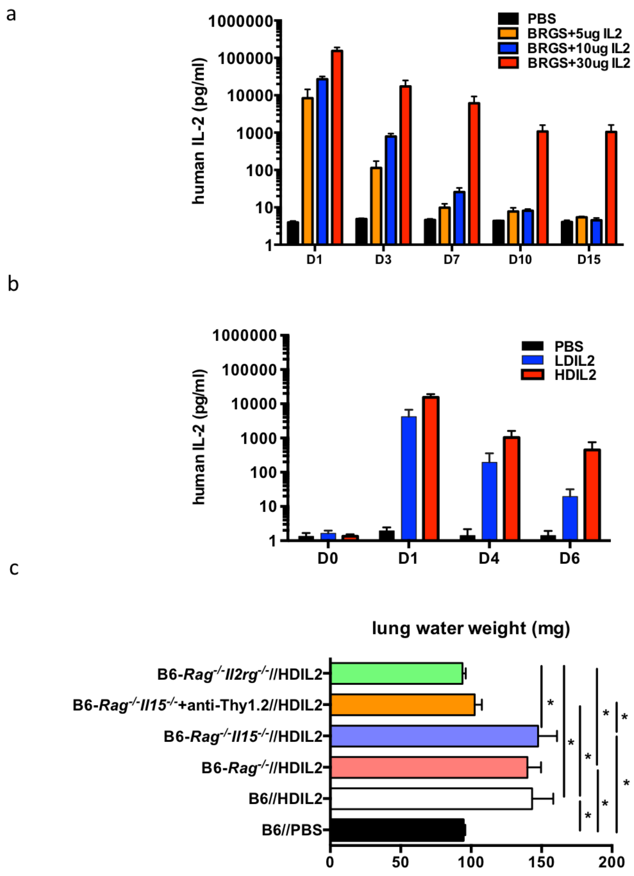

Supplementary Figure 1. Serum levels of IL-2 after hydrodynamic injection in BALB/c*Rag2*$^{-/-}$*Il2rg*$^{-/-}$*Sirpa*^{NOD} and human immune system mice. 10 to 14 week old mice were hydrodynamically injected with PBS only or indicated doses of human IL-2 encoding plasmids. Sera were collected at indicated days post injection. (a, b) Concentrations ± SEM of human IL-2 in sera after hydrodynamic injection in BALB/c*Rag2*$^{-/-}$*Il2rg*$^{-/-}$*Sirpa*^{NOD} (BRGS) mice (n=5 per group) (a) and in human immune system mice (n=4 per group) (b). (c) Pulmonary edema from indicated mouse strains and treatment groups were measured by lung water weight. Lung water weight was calculated by subtracting weights of dried lungs from that of wet lungs. Data are pooled from 2 independent experiments, 1-way ANOVA test with a Tukey test.

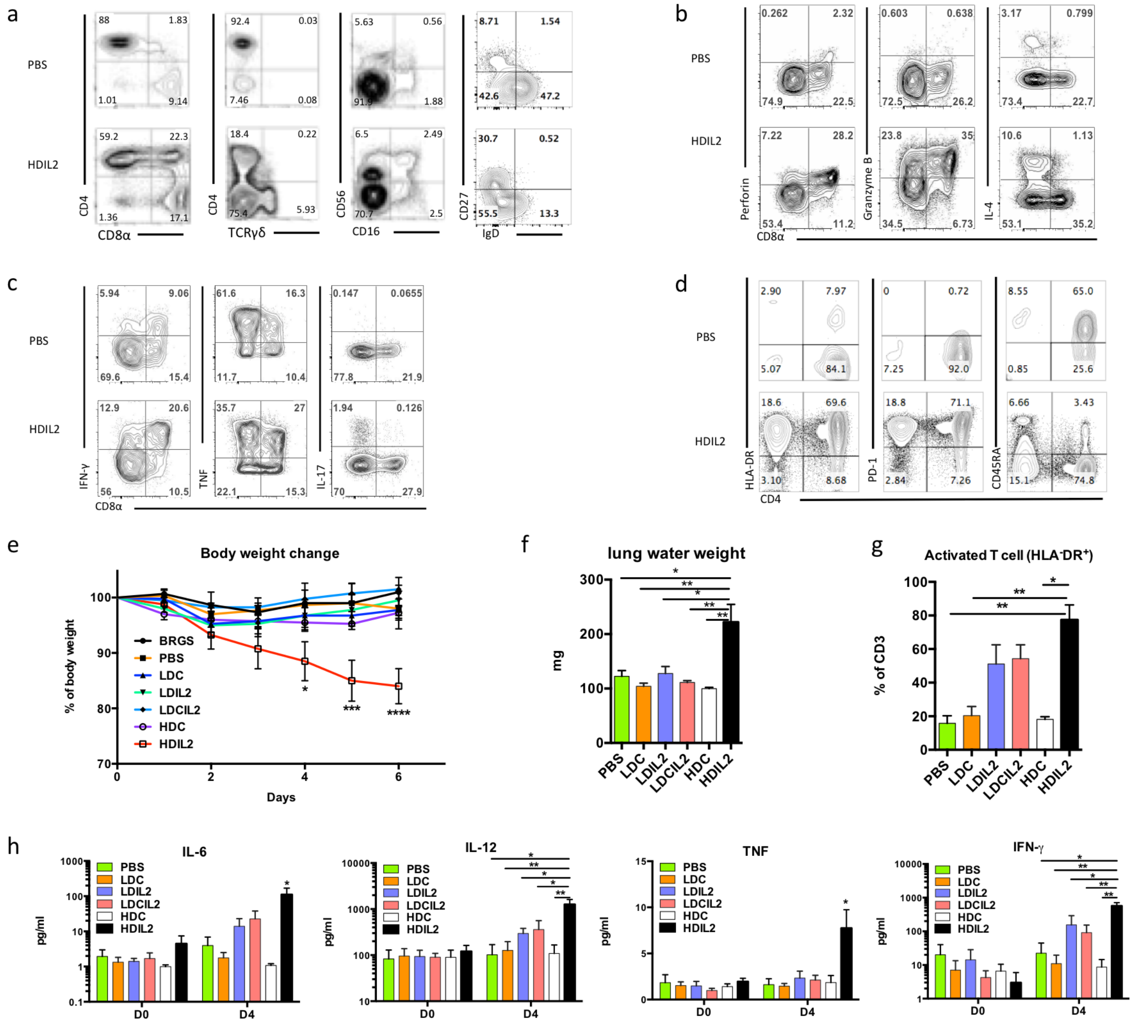

Supplementary Figure 2. **Characterization of human immune responses in low-dose IL-2 and high-dose IL-2 human immune system mice**. (a-d) 10 to 14 week old human immune system (HIS) mice treated with PBS, low-dose IL-2 (LDIL2) or high-dose IL-2 (HDIL2) were sacrificed at day 6 post injection. (a) Representative flow cytometric data showing the $CD4^+$, $CD8\alpha^+$, $TCR\gamma\delta^+$ T cell percentages in $CD3^+$ T cells, CD16/CD56 expression in $CD45^+$ cells, and CD27/IgD expression in $CD19^+$ B cells from spleens of indicated groups. (b) Flow cytometric data showing perforin, granzyme B and IL-4 expression within splenic $CD45^+CD3^+$ human T cells after 4 hr culture *ex vivo* with Golgiplug. (c) Flow cytometric data showing IFN-γ, TNF and

IL-17 within splenic $CD45^+CD3^+$ human T cells after 4 hr culture *ex vivo* with Golgiplug and PMA/ionomycin. Data are representative of 2 independent experiments. (d) Representative flow cytometric data showing HLA-DR, PD-1 and CD45RA expression within splenic $CD45^+CD3^+$ human T cells. (e-h) 10 to 14 week old HIS mice (n=4 for PBS group, n=6 for HDIL2 group, and n=5 for other groups) treated with PBS, LDIL2, HDIL2 or additional control groups with empty plasmids were sacrificed and sera were collected at day 6 post injection. Data are pooled from 2 independent experiments. (e) Percentages of body weight change after hydrodynamic injection in HIS mice. Comparisons are between non-treated BRGS HIS mice and the other groups by repeated-measures two-way ANOVA with a Sidak test. (f) Pulmonary edema of indicated groups were measured by lung water weight. Lung water weight was calculated by subtracting weights of dried lungs from that of wet lungs. Data are analyzed by 1-way ANOVA test with a Tukey test. (g) HLA-DR expression on splenic $CD3^+$ T cells from indicated groups. Data are analyzed by 1-way ANOVA test with a Tukey test. (h) Serum cytokine levels of IL-6, IL-12. TNF and IFN-γ were analyzed. Data are analyzed by 1-way ANOVA test with a Tukey test. *$P<0.05$, **$P<0.01$, ***$P<0.001$, ****$P<0.0001$.

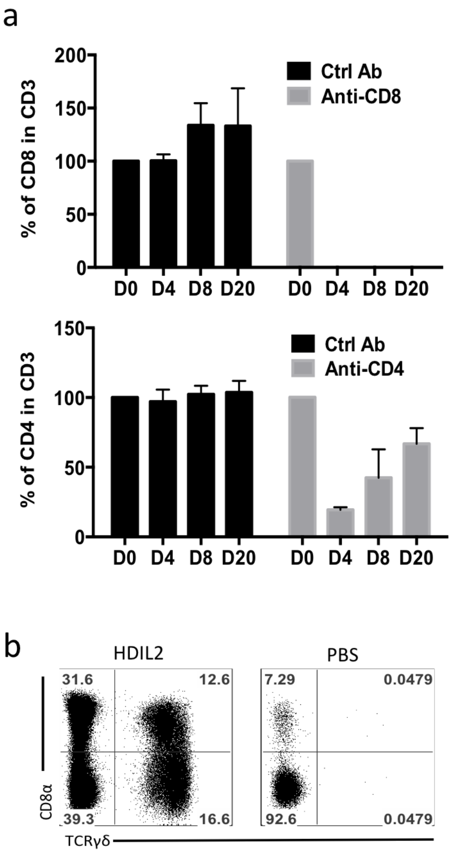

Supplementary Figure 3. **Kinetics and effects of CD4$^+$ and CD8$^+$ T cell depletion in high-dose IL-2 human immune system mice.** (a) Single dose of 200μg anti-CD4 or anti-CD8 depleting antibodies were i.v. injected in 10 to 14 week old human immune system (HIS) mice (n=4 per group). Mice were bled at indicated dates to check for the percentages of CD4$^+$ and CD8$^+$ T cells within CD45$^+$CD3$^+$ cells and the percentages were normalized to D0. (b) Representative flow cytometric data showing CD8α and TCRγδ expression within CD45$^+$CD3$^+$ human T cells in spleen of PBS treated and high-dose IL-2 (HDIL2) treated HIS mice.

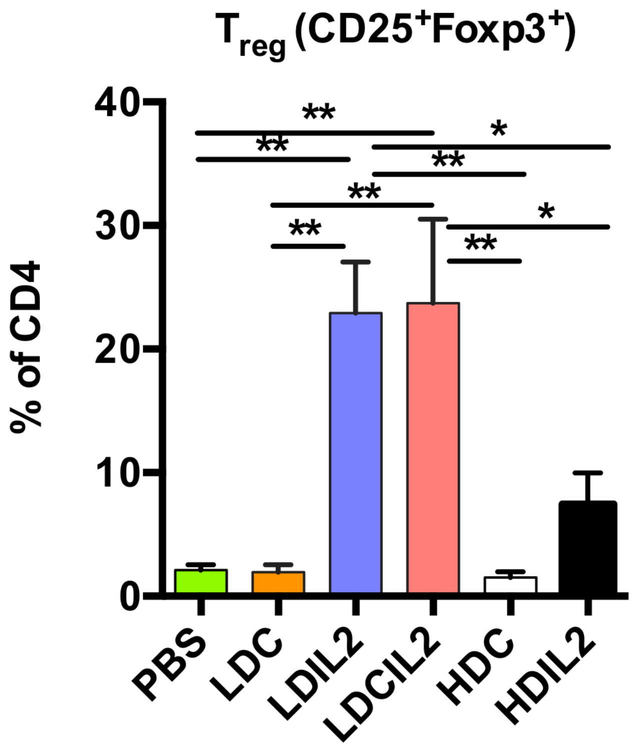

Supplementary Figure 4. Regulatory T cell expansion is IL-2 dose-dependent. 10 to 14 week old human immune system mice treated with PBS, low-dose IL-2 (LDIL2), high-dose IL-2 (HDIL2) or additional control groups with empty vectors were sacrificed and sera were collected at day 6 post injection mice (n=4 for PBS group, n=6 for HDIL2 group, and n=5 for other groups). Figure shows percentages of Foxp3^{+} Regulatory T (T_{reg}) cells within splenic CD45^{+}CD3^{+}CD4^{+} T cells. Data are pooled from 2 independent experiments. Data are analyzed by 1-way ANOVA test with a Tukey test. *P<0.05, **P<0.01.

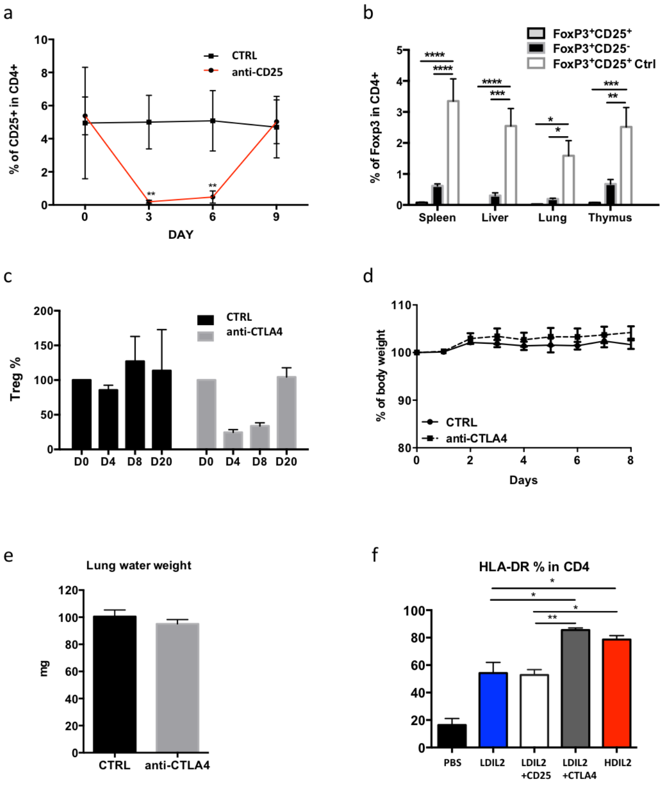

Supplementary Figure 5. Kinetics and effects of CD25 and CTLA-4 depletion in low-dose IL-2 human immune system mice. Single dose of 200µg anti-CD25, anti-CTLA-4, or control mAb were i.v. injected to 10 to 14 week old human immune system (HIS) mice (n=4 per group). (a, c) Mice were bled at indicated dates to check for the percentages of Foxp3$^+$ cells within CD45$^+$CD3$^+$CD4$^+$ T cells and the percentages were compared to day 0. (b) the percentages of subsets of Foxp3$^+$ cells within CD45$^+$CD3$^+$CD4$^+$ T cells from indicated organs of anti-CD25 treated or control mAbs treated HIS mice. (d, e) 14 week old HIS mice were injected twice with 300µg anti-CTLA-4 mAbs at D0 and D4 (n=5 per group). Body weight change was measured daily and normalized to D0. Mice were sacrificed at D8. Pulmonary edema was measured by lung water weight. Lung water weight was calculated by subtracting weights of dried lungs from that of wet lungs. (f) HLA-DR expression on CD4$^+$ T cells from indicated groups. *P<0.05, **P<0.01, by 1-way ANOVA test with a Tukey test.

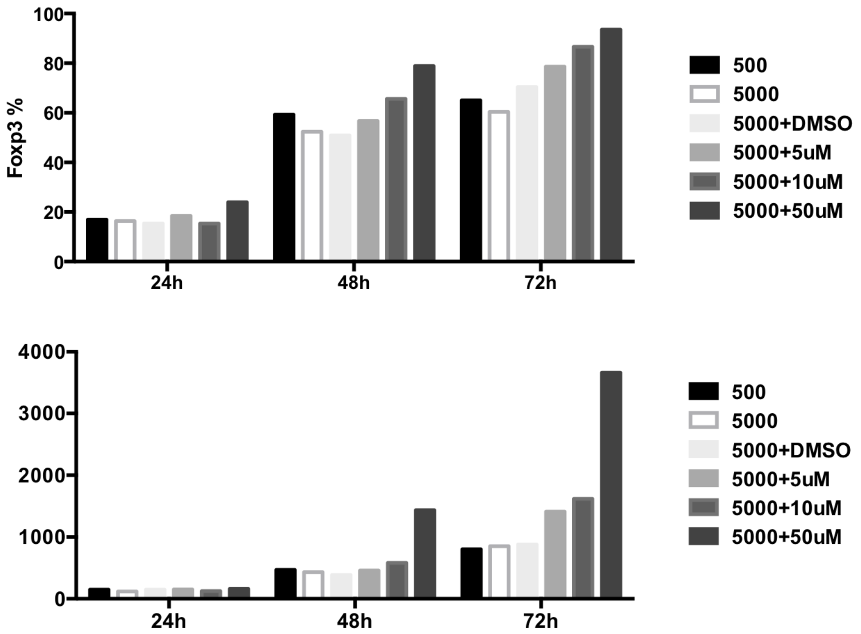

Supplementary Figure 6. **Kaempferol stabilizes Foxp3 expression in cultured human regulatory T cells.** Human $CD4^+$ T cells were purified from cord blood and cultured in X-vivo medium supplemented with 50U/ml IL-2 for 1-2 days following activation by Dynabeads in the presence of increasing doses of IL-2 with or without Kaempferol. Samples were collected at 24h, 48h, and 72h, followed by FACS analysis for percentages of $Foxp3^+$ within CD4 T cells and MFI of $Foxp3^+$ CD4 T cells. Data are a representative dataset of 2 independent experiments.

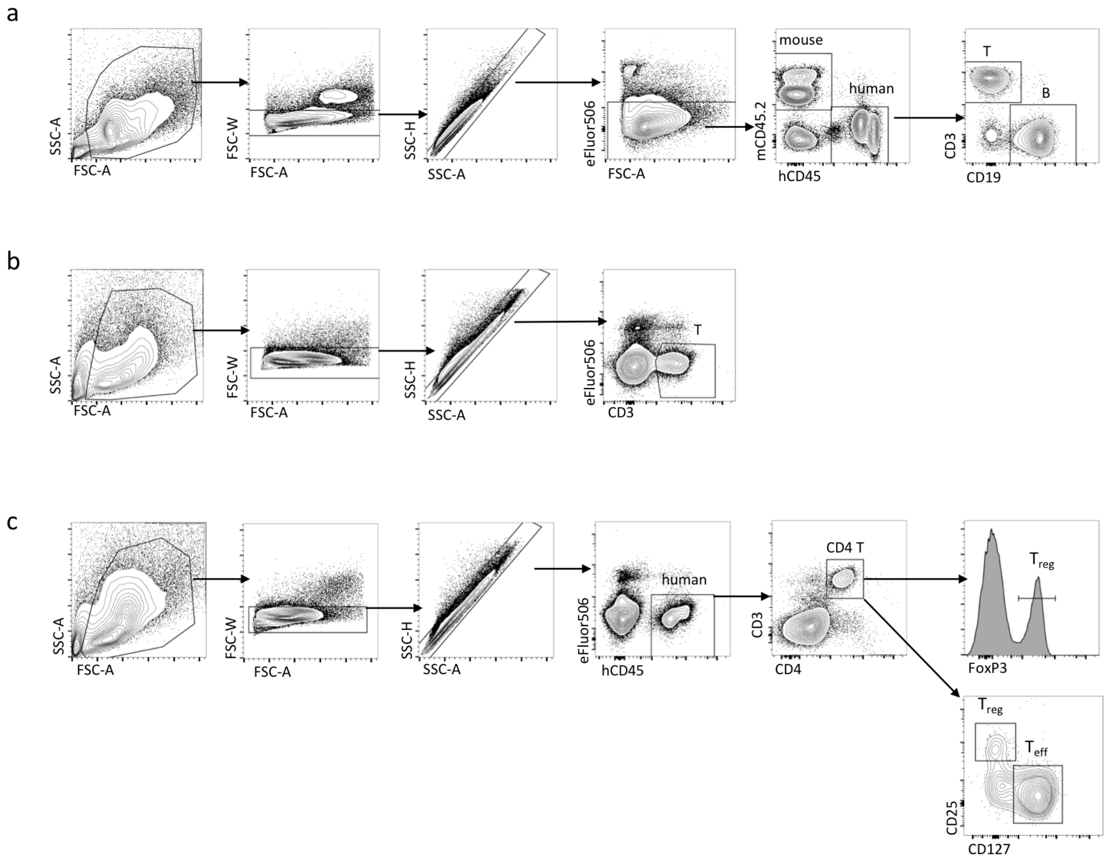

Supplementary Figure 7. Gating strategies for flow cytometry and cell sorting. (a) Gating strategy of human immune subsets in human immune system (HIS) mice for analysis presented in Fig. 2b, Fig. 3a and Supplementary Fig. 2a and Fig. 3. (b) Gating strategy of human CD3^{+} T cells for analysis of cytokine production, transcriptional factors and activation presented in Fig. 2c, d and Supplementary Fig. 2b-d. (c) Gating strategy of human regulatory T (T_{reg}) cells for flow cytometry analysis presented on Fig. 4-7 and supplementary Fig. 4-6, or cell sorting presented in Fig. 4f.

Supplemental Table 1. Antibodies used for FACS analysis.

Antigen	Reactivity	Clone	Supplier	Dilution (50µl)
CD45	Human	HI30	BD	2.5µl
CD45.2	Mouse	104	Biolegend	1µl
CD3	Human	UCHT1	BD	2.5µl
CD4	Human	RPA-T4	BD	2.5µl
CD8α	Human	BW135/80	Miltenyi	2.5µl
TCRγδ	Human	11F2	Miltenyi	2.5µl
CD56	Human	B159	BD	2.5µl
CD16	Human	3G8	BD	2.5µl
Perforin	Human	dG9	eBioscience	3µl
IFN-γ	Human	B27	BD	3µl
HLA-DR	Human	L243	Biolegend	2.5µl
PD-1	Human	EH12.2H7	Biolegend	2.5µl
CD45RA	Human	T6D11	Miltenyi	2.5µl
CD25	Human	BC96	Biolegend	2.5µl
CD127	Human	eBioRDR5	eBioscience	2.5µl
CD45RO	Human	UCHL1	Miltenyi	2.5µl
CD120b	Human	3G7A02	Biolegend	2.5µl
CTLA-4	Human	L3D10	Biolegend	2.5µl
CD19	Human	HIB19	BD	2.5µl
CD27	Human	M-T271	Miltenyi	2.5µl
FOXP3	Human	PCH101	eBioscience	3µl
T-BET	Human	4B10	Biolegend	3µl
EOMES	Human	WD1928	Ebioscience	3µl
